# Rumen-derived lipopolysaccharide provoked inflammatory injury in the liver of dairy cows fed a high-concentrate diet

**DOI:** 10.18632/oncotarget.18151

**Published:** 2017-05-24

**Authors:** Junfei Guo, Guangjun Chang, Kai Zhang, Lei Xu, Di Jin, Muhammad Shahid Bilal, Xiangzhen Shen

**Affiliations:** ^1^ College of Veterinary Medicine, Nanjing Agricultural University, Nanjing, P.R. China

**Keywords:** subacute ruminal acidosis, lipopolysaccharide, inflammatory injury, liver, dairy cows, Immunology and Microbiology Section, Immune response, Immunity

## Abstract

Rumen-derived lipopolysaccharide (LPS) is translocated from the rumen into the bloodstream when subacute ruminal acidosis (SARA) occurs following long-term feeding with a high-concentrate (HC) diet in dairy cows. The objective of this study was to investigate the mechanism of inflammatory responses in the liver caused by HC diet feeding. We found that SARA was induced in dairy cows when rumen pH below 5.6 lasted for at least 3 h/d with HC diet feeding. Also, the LPS levels in the portal and hepatic veins were increased significantly and hepatocytes were impaired as well as the liver function was inhibited during SARA condition. Meanwhile, the mRNA expression of immune genes including TNF receptor associated factor 6 (TRAF6), nuclear factor-kappa B (NF-κB), p38 mitogen-activated protein kinase (MAPK), extracellular regulated protein kinases (ERK) MAPK, Interleukin-1 (IL-1) and serum amyloid A (SAA) in the liver were significantly increased in SARA cows. Moreover, the phosphorylation level of NF-κB p65 and p38 MAPK proteins in the liver and the concentration of Tumor Necrosis Factor (TNF-α), Interleukin-1β (IL-1β) and Interleukin-6 (IL-6) in peripheral blood were obviously increased under SARA condition. In conclusion, the inflammatory injury in the liver caused by LPS that traveled from the digestive tract to the liver through the portal vein after feeding with a HC diet.

## INTRODUCTION

In order to increase the milk production, dairy cows are always fed high-concentrate (HC) diet in dairy industry. However, HC diets can depress rumen pH and cause subacute ruminal acidosis (SARA) in these cows [1]. SARA is considered as the most economically important form of acidosis in dairy farm, which is confirmed once the rumen pH value drops below 5.6 and lasts for more than 3 hours per day [2–4]. Thus, measuring rumen pH is one of the greatest impediments to the diagnosis of SARA [3, 5]. A number of signs of SARA in dairy cows, including inflammation, acute phase responses, laminitis, and liver abscesses have been attributed to the translocation of free lipopolysaccharides (LPS) in gram-negative bacteria from the digestive tract into the interior circulation [6]. It has been suggested that SARA induced by feeding high-grain diets in dairy cows could increase their free ruminal LPS [6].

LPS is a part of the gram-negative bacteria cell wall, which contributes to the permeability barrier of gastrointestinal tract [6, 7]. When cell proliferation and disintegration of gram-negative bacteria occur in large quantities because of the action of bacteria autolytic enzyme during SARA, LPS are released from the cell wall and the toxicity increases dramatically [6, 8, 9]. Consequently, ruminal free LPS are translocated into the blood stream through the portal vein. One of the vitro studies showed that LPS translocated across the rumen and the colon wall [10]. Meanwhile, there are also some studies found that the rumen epithelium has low permeability to LPS [11]. However, grain-induced SARA could increase the concentration of free ruminal LPS. Moreover, by-pass starch could be able to interrupt the barrier function of the monolayer epithelium of the hindgut, causing sophisticated systemic inflammatory response [11]. It has been indicated that LPS is implicated in metabolic disorders such as laminitis, ruminal acidosis and sudden death syndrome that are related to HC diet [6, 12].

LPS is a highly potent activator of the pro-inflammatory responses, which is mediated by the toll-like receptor 4 (TLR-4) signaling pathway [13]. LPS can combine with LPS-binding protein (LBP) and transferred to the cell surface with the help of cluster of differentiation 14 (CD14) [14, 15]. Then the complex of LPS-LBP-CD14 activates TLR-4 signaling pathway and facilitates the secretion of pro-inflammatory cytokines such as Tumor Necrosis Factor (TNF-α), interlukine-1 (IL-1) and IL-6, which can cause an inflammatory reaction by activating the expression of receptors in hepatocytes and other target cells [6, 16, 17]. Systemic consequences such as leukocytosis and changes of acute phase proteins (APPs) in peripheral blood have been observed. In other words, LPS increases the concentration of LPS-binding protein (LBP), serum amyloid A (SAA) and haptoglobin (HP) in peripheral blood during SARA [3, 11].

Liver plays an important role in natural immune response and is considered to be the major contributor to the APP production in plasma [18]. Part of the LPS circulated in gastrointestinal tract results from the high grain diet is transported via portal vein to the liver where rich in hepatocytes and Kupffer cells [19]. An *in vitro* experiment used Kupffer cells (liver macrophages) in cows showed that liver made a great contribution to cytokines production [18, 20]. Hepatocytes can excrete endotoxins present in the circulatory system in the bile, and detoxify LPS through the activity of Kupffer cells [20]. Some studies proved that nutrient metabolism in the liver is inhibited and the immune function of the liver is reduced in the presence of strong endotoxins. For instance, endotoxemia is caused by an excess of LPS translocated into the liver since liver is one of the main target organs of LPS infusion [21].

However, little investigation has been conducted on fully understanding of the inflammatory damages in the liver because of endogenous LPS from the digestive tract as a result of high concentrate feeding. Therefore, this study aimed to detect the mechanism of the liver inflammatory responses caused by endogenous LPS. We hypothesized that LPS derived from the digestive tract to the liver via the portal vein during SARA can cause hepatocyte impairment, inhibit the liver function and activate hepatic immune-related gene expression as well as inflammatory signaling pathway.

## RESULTS

### Ruminal pH and the concentration of LPS in the portal and hepatic veins

**Figure 1:**
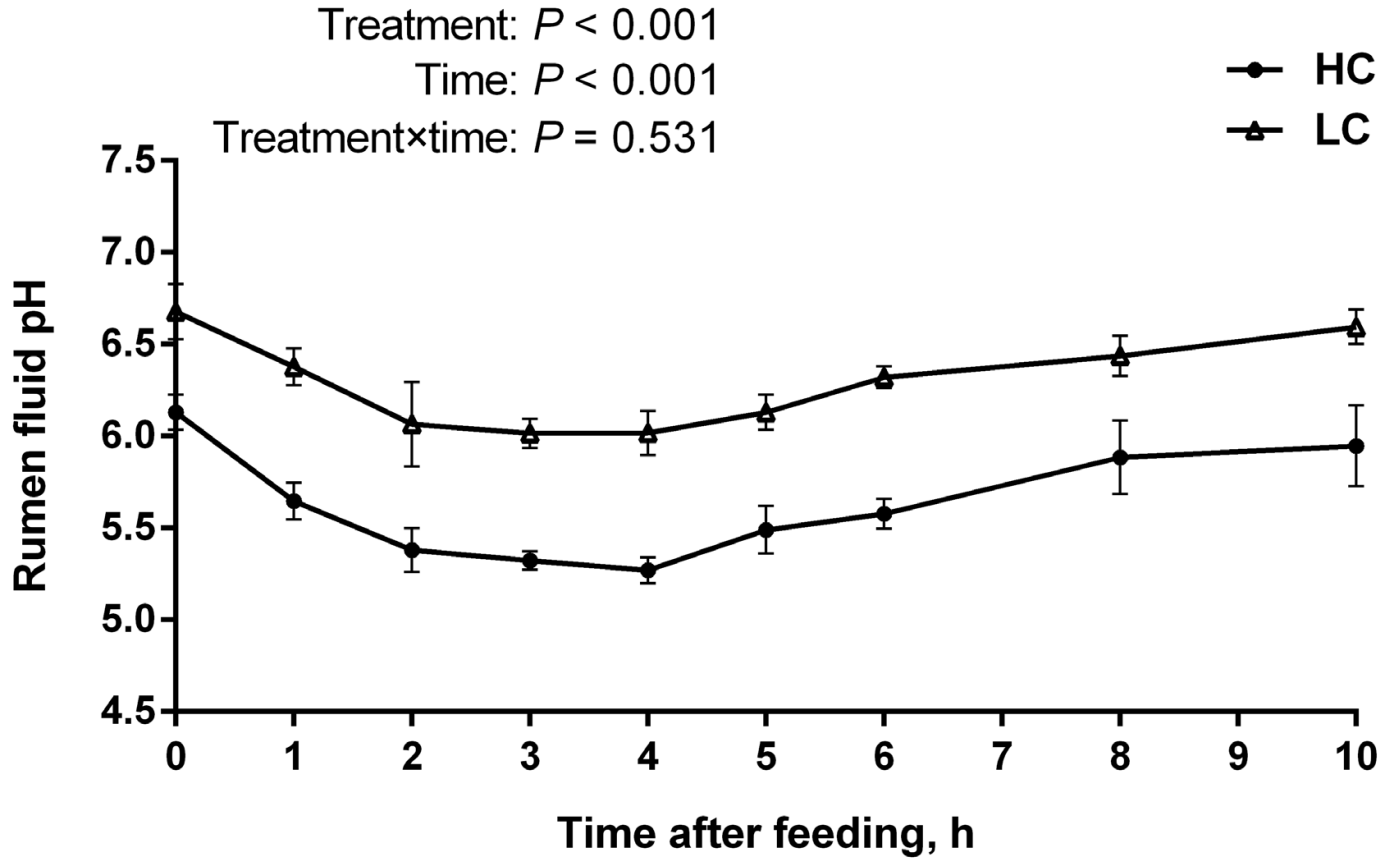
pH value of rumen fluid of high-concentration (HC) and low-concentrate (LC) diet Rumen pH value was measured at different time points post feeding. Rumen pH decreased dramatically in HC group compared to LC group (mean ± SEM, *n* = 6). Differences between two groups were considered as significant when *P <* 0.05.

The data showed that rumen pH in the HC group was much lower than that in the low-concentrate (LC) group (*P* < 0.01) (Figure 1). The duration for rumen pH value below 5.6 in the HC group was more than 3 hours (180 min) per day while LC group was not, indicating that SARA was successfully induced by HC diet. Besides, rumen pH value dropped to the lowest at 4 h post feeding.

**Figure 2:**
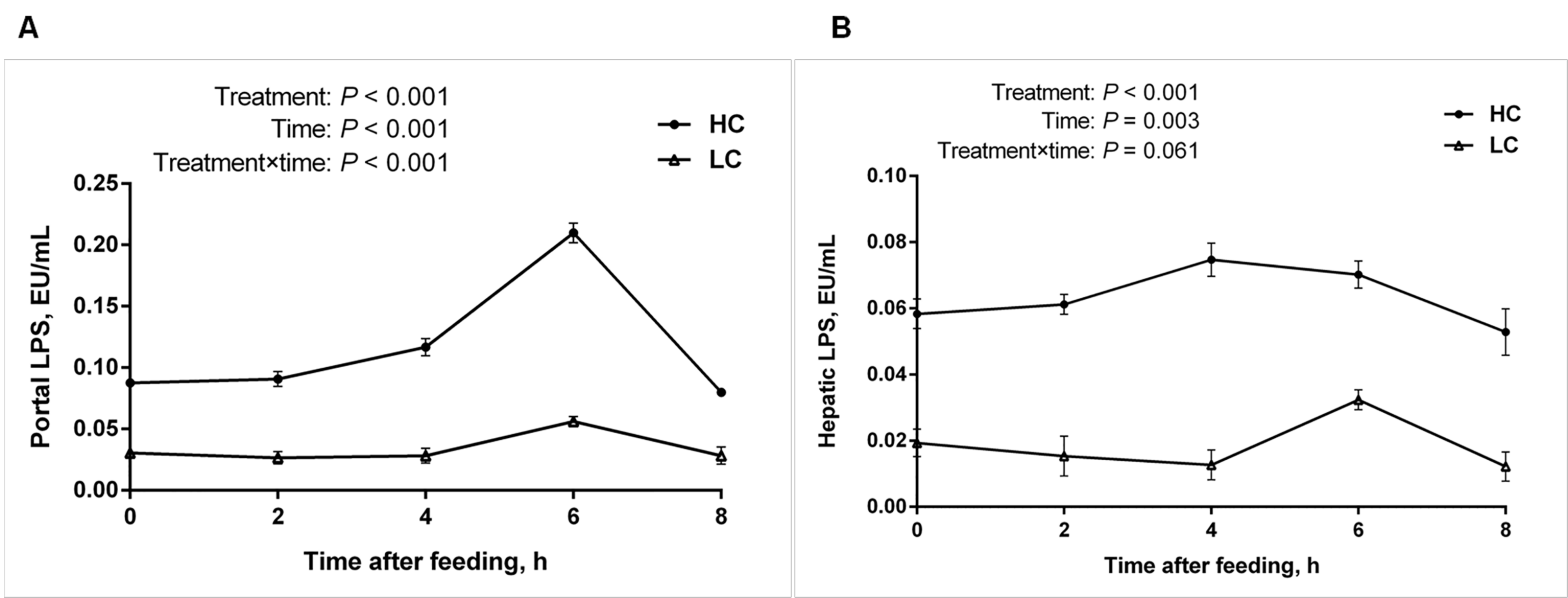
The concentration of LPS in portal and hepatic veins at different time points post feeding in the liver of dairy cows from high-concentrate (HC) and low-concentrate (LC) diet **A**. LPS concentration in portal vein (mean ± SEM, *n* = 6). **B**. LPS concentration in hepatic vein (mean ± SEM, *n* = 6). LPS concentration in HC group was much higher than LC group. Difference was considered as significant between two groups when *P <* 0.05.

The LPS levels in portal vein (Figure 2A) and hepatic vein (Figure 2B) were significantly increased in HC group compared with the LC group (*P* < 0.01), and the LPS concentration reached the highest level at 6 h post feeding in portal vein (Figure 2A). In addition, the LPS concentration was higher in the portal vein than in the hepatic vein in both groups, indicating the scavenging activity of LPS in the liver.

### Histopathologic changes in the liver

**Figure 3:**
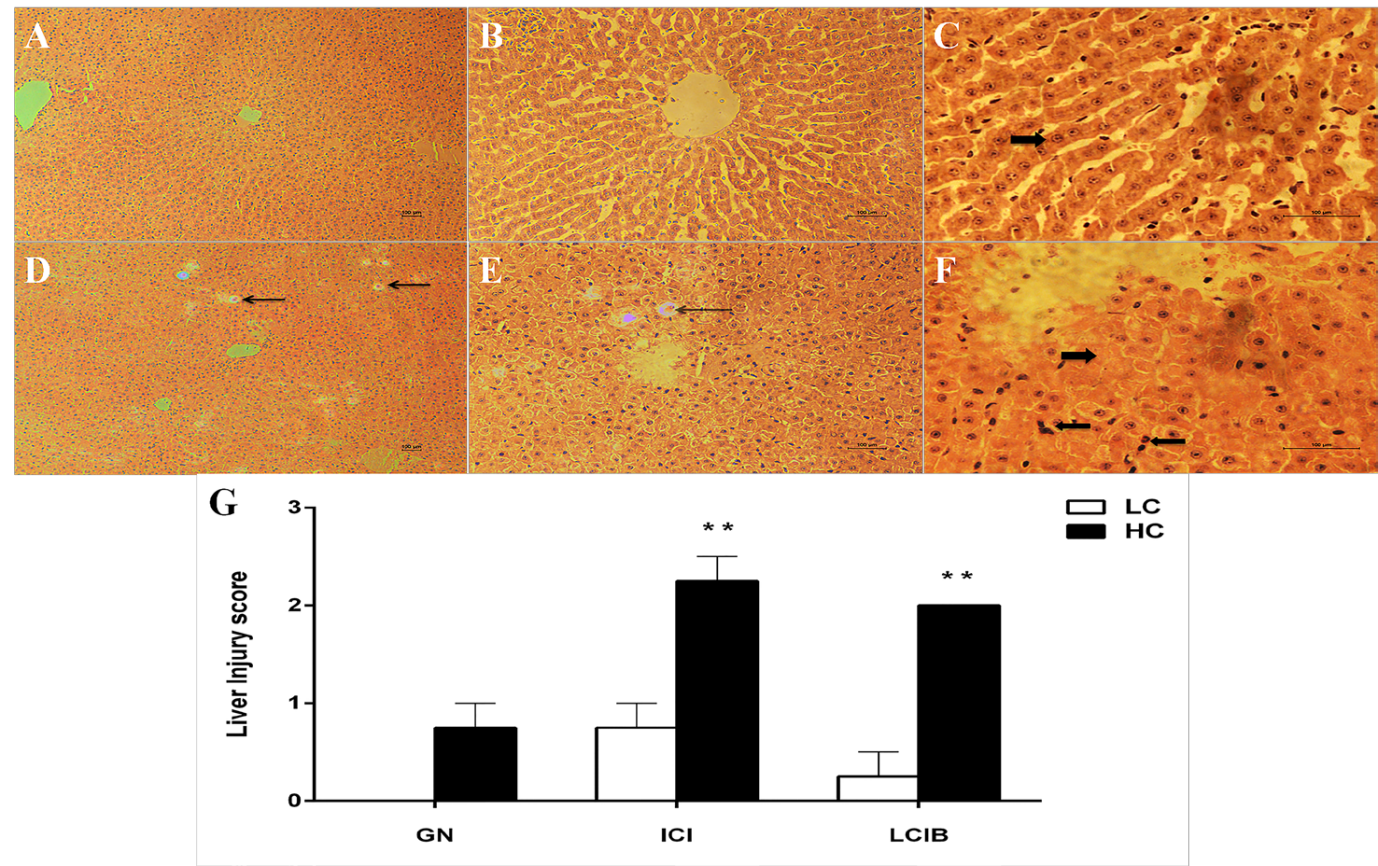
The effect of high-concentrate (HC) diet on hepatocytes histopathologic changes in dairy cows Representative photomicrographs with hematoxylin and eosin staining. HC: High-concentrate diet. LC: Low-concentrate diet. **A**., **B**. and **C**. LC group. **D**., **E**. and **F**. HC group. **G**. Hepatocytes injury score. All the sections were observed at 100, 200 or 400 × magnification. GN: Glycogenated nuclei. ICI: Inflammatory cells infiltrate. ICIB: Liver cells injury ballooning. The thin arrow indicates GN damages; thick right arrow in F indicates ICIB damages while in C indicates no disruption; thick left arrow indicates inflammatory cells infiltrate. The damage score are expressed as the mean ± SEM and asterisks indicate the significant difference between two groups (**P < 0.01).

Histopathologic changes in the liver tissues from both groups were examined with hematoxylin and eosin staining. The photomicrographs are showed in Figure 3. Glycogenated nuclei (Figure 3D and 3E), inflammatory cells infiltration and liver cells injury ballooning (Figure 3E and 3F) were observed in HC group while no obvious disruption was founded in LC group (Figure 3A, 3B and 3C). The hepatocytes injury score was significantly higher in HC group than that in LC group (Figure 3G, *P* < 0.01).

### Biochemical parameters

The changes of biochemical parameters in each group are presented in Table 3. Albumin (ALB) and total protein (TP) concentrations in the peripheral blood of the HC group were significantly lower than that in the LC group (*P* < 0.05), aspartate aminotransferase (AST), Alanine aminotransferase (ALT) and Lactate dehydrogenase (LDH) activity were significantly higher compared to LC group (*P* < 0.01). These findings demonstrated that the hepatocytes were injured and the liver function was inhibited by HC feeding.

**Table 1:** Ingredients, nutrient composition and forage-to-concentrate radio (F:C) of the diets used in this experiment

Item	Diet	
	LC	HC
Ingredient, %DM basis		
Corn silage	30	20
Alfalfa hay	30	20
Corn	24.3	32
Bran	0	12.4
Soybean meal	13.5	13
Calcium phosphate dibasic	0.85	0.45
Powder	0	0.8
Salt	0.35	0.35
Premix^a^	1	1
F:C	6:4	4:6
Nutrient composition^b^		
NE, MJ/kg	6.36	6.71
CP, %DM	16.99	16.92
EE, %DM	3.93	4.07
NDF, %DM	36.54	31.45
ADF, %DM	22.51	17.56
NFC, %DM	39.32	33.76

^a^ Premix contained 5.25 g/kg of Fe, 1.2 g/kg of Cu, 5.5 g/kg of Mn, 13 g/kg of Zn, 50mg/kg of Co, 27 mg/kg of Se, 170 mg/kg of I, 1,900 ku/kg of vitamin A, 250 ku/kg of vitamin D, and 3 g/kg of vitamin E;

^b^ Nutrient composition: NE = Net energy; CP = Crude protein; EE = Ether extract; NDF = Neutral detergent fiber; ADF = Acid detergent fiber; NFC = Non-fibrous carbohydrates.

**Table 2:** Primer sequences for reverse transcription and RT-qPCR

Gene	Forward primer	Reverse primer	PCR products (bp)
TLR-4	GGACCCTTGCGTACAGGTTG	GGAAGCTGGAGAAGTTATGGC	244
TRIF	AAGACACCGCTTACCTGTCG	AGGGGTCGATGGAGAAGGAA	113
TRAF6	CGGTGACTCTCTCCAGCTCT	TGGACATTTGTGACCTGCAT	194
TAK1	TGTGTAGTGCCAGTGGAAAGG	AGATGCTGGCTTCACAGAGT	104
IKK	GAAGAGTGAGGACCTGGTGGC	AGCTCCAGTCTAGAGACCTCAAA	113
IκB	AAGGTGGAAATGGCCCTCAG	ATCCTACAAGGGACCGAGCA	136
NF-κB	ATACGTCGGCCGTGTCTAT	GGAACTGTGATCCGTGTAG	129
p38	CTGCCGCCTGGCATATGTTT	TGCTTTTCCCACCCACATTGA	128
ERK	AACAAAGTCCGAGTCGCCAT	CGATGGTCGGTGCTCGAATA	148
JNK1	ACAGGGTGCATTAATTTTATTAGGC	AGCTAGTTAACTGTCAAGCTAAAGA	144
JNK2	ACAGCGTGCACCAACTTTATG	CTCTGGCTTGACTTGCGTTCT	109
IL-1β	GGCCAAAGTCCCTGACCTCT	CTGCCACCATCACCACATTC	167
IL-6	GGAGGAAAAGGACGGATGCT	GGTCAGTGTTTGTGGCTGGA	227
IL-10	GTGGAGAAGGTGAAGAGAGTC	GTGGGAGCTGAGGTATCAGAG	296
TNF-α	CTTCTGCCTGCTGCACTTCG	CTGTGAGTAGATGAGGTAAAGC	271
SAA	CTTTCCACGGGCATCATTTT	GCCAGCAGGTCTGAAGTGG	170
Hp	ACAAGGACCATTGGACAGCAACT	GGCATCCAATGAGCCACCGAT	282
LBP	GCAAGATCACTGGATTCTTGGA	AAAACAGGAAGTCCTTGTGGATC	228
GAPDH	CATGTTCCAGTATGATTCCACCC	GAGCTTCCCGTTCTCTGCC	177

**Table 3:** Plasma levels of liver function indicators in dairy cows from high-concentration (HC) and low-concentrate (LC) diet

Item	LC	HC	SEM^a^	*P*-Value
TP, g/L	79.50	70.33	2.15	0.03
ALB, g/L	31.83	29.08	0.68	0.04
AST, IU/L	32.83	49.50	3.07	<0.01
ALT, IU/L	16.17	21.08	1.01	<0.01
LDH, IU/L	896	1241.90	62.20	<0.01

Biochemical parameters of liver function were determined. TP = Total protein. ALB = Albumin. AST = Aspartate aminotransferase. ALT = Alanine aminotransferase. LDH = Lactate dehydrogenase. Difference was considered as significant when *P* < 0.05. ^a^SEM = Standard error of means.

### mRNA expression of immune-related genes in the liver

**Figure 4:**
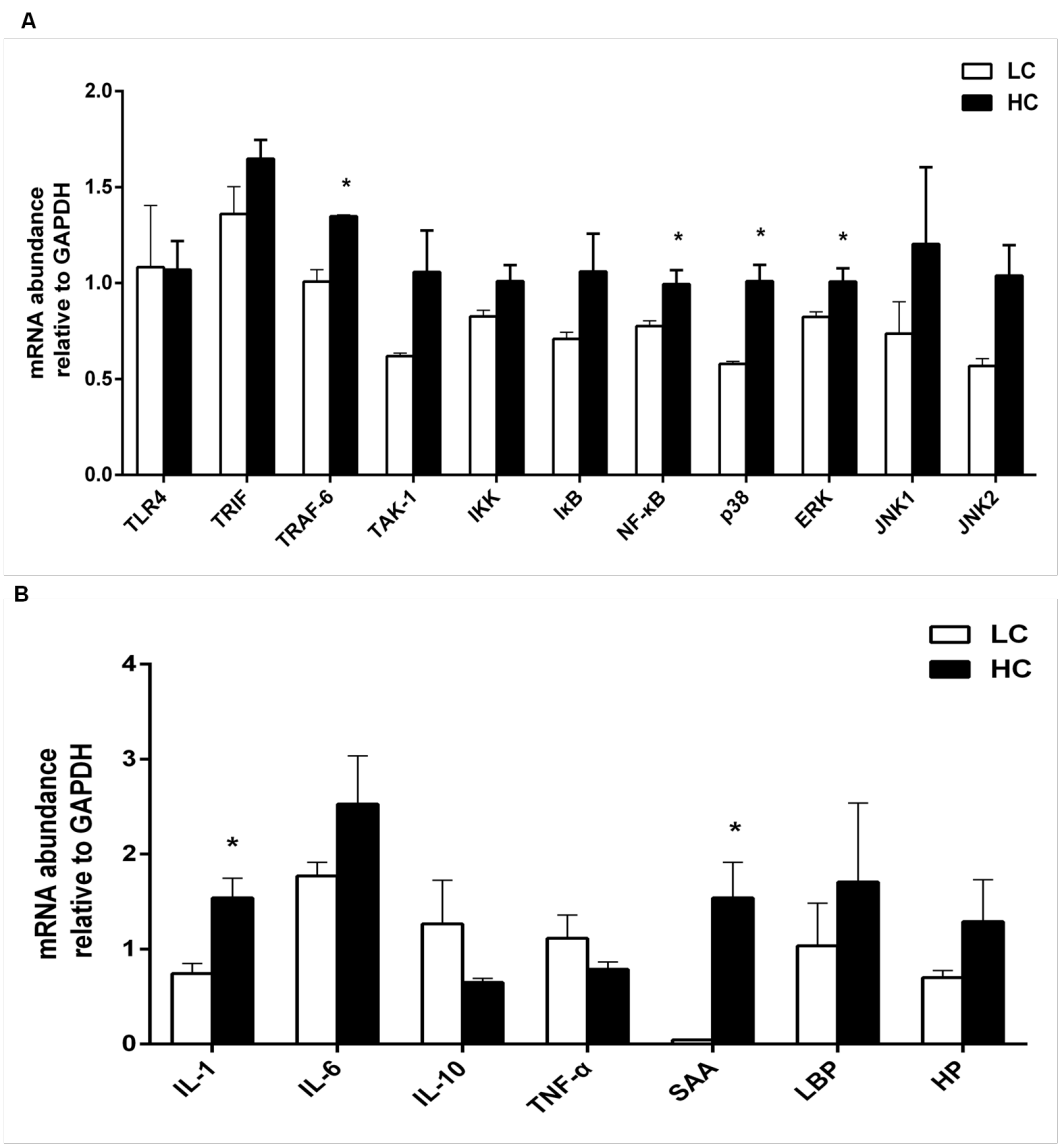
The mRNA relative expression of immune genes in the liver of dairy cows from high-concentrate (HC) and low-concentrate (LC) diet detected by Real-Time qPCR **A**. Immune genes involved in inflammatory signaling pathway. The expression of TRAF6, NF-κB-p38 MAPK and ERK MAPK in the liver were increased in HC group compare to LC group. **B**. Pro- and anti-inflammatory cytokines and acute phase proteins. The expression of IL-1βand SAA in the liver were obviously higher in HC group than LC group. The data are expressed as the mean ± SEM and asterisks indicate the differences between two groups (*P < 0.05, *n* = 6).

The relative quantitative expression of immune-related genes in the liver is shown in Figure 4. The mRNA relative expression of immune genes involved in inflammation such as TNF receptor associated factor 6 (TRAF6), nuclear factor-kappa B (NF-κB), p38 mitogen-activated protein kinase (MAPK), extracellular regulated protein kinases (ERK) MAPK was increased by HC feeding (Figure 4A, *P <* 0.05), as well as pro-inflammatory cytokine IL-1 and acute phase protein SAA (Figure 4B, *P* < 0.05). However, the mRNA expression of TLR-4 was lower in the HC group than LC group with no significant difference between the two groups.

### Phosphorylation levels of NF-κB p65 and p38 MAPK proteins

**Figure 5:**
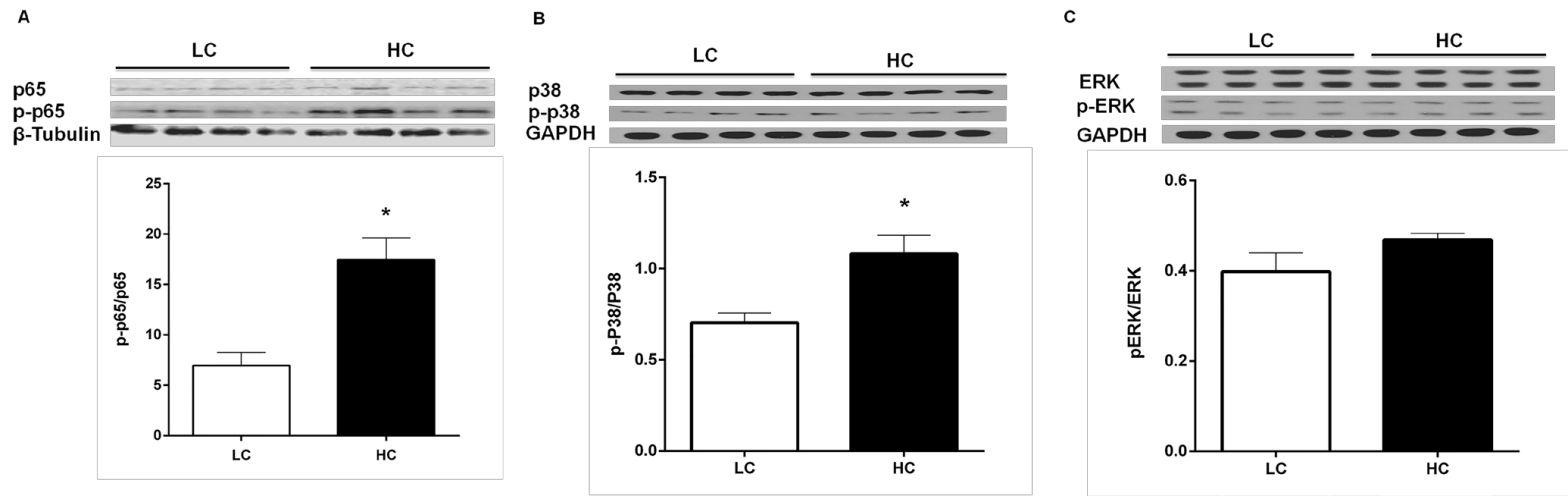
NF-κB p65 **A**., p38 **B**. MAPK and ERK MAPK **C**. phosphorylation levels in the liver of dairy cows from high-concentrate (HC) and low-concentrate (LC) diet. The phosphorylation level of NF-κB and p38 MAPK in the liver were significantly higher in HC group than LC group. The data are expressed as the mean ± SEM, and the asterisks indicate the significant differences between two groups (*P < 0.05, *n* = 6).

The phosphorylation levels of NF-κB p65, p38 MAPK and ERK MAPK were performed by western blot analysis. Compared to LC group, NF-κB p65 (Figure 5A) and p38 MAPK (Figure 5B) phosphorylation levels were markedly higher in the liver in the HC group (*P* < 0.05), indicating that NF-κB p65 and p38 MAPK were activated in the liver. But there was no significant difference between two groups of ERK MAPK phosphorylation level (Figure 5C).

### Concentration of pro-inflammatory cytokines in peripheral blood

**Figure 6:**
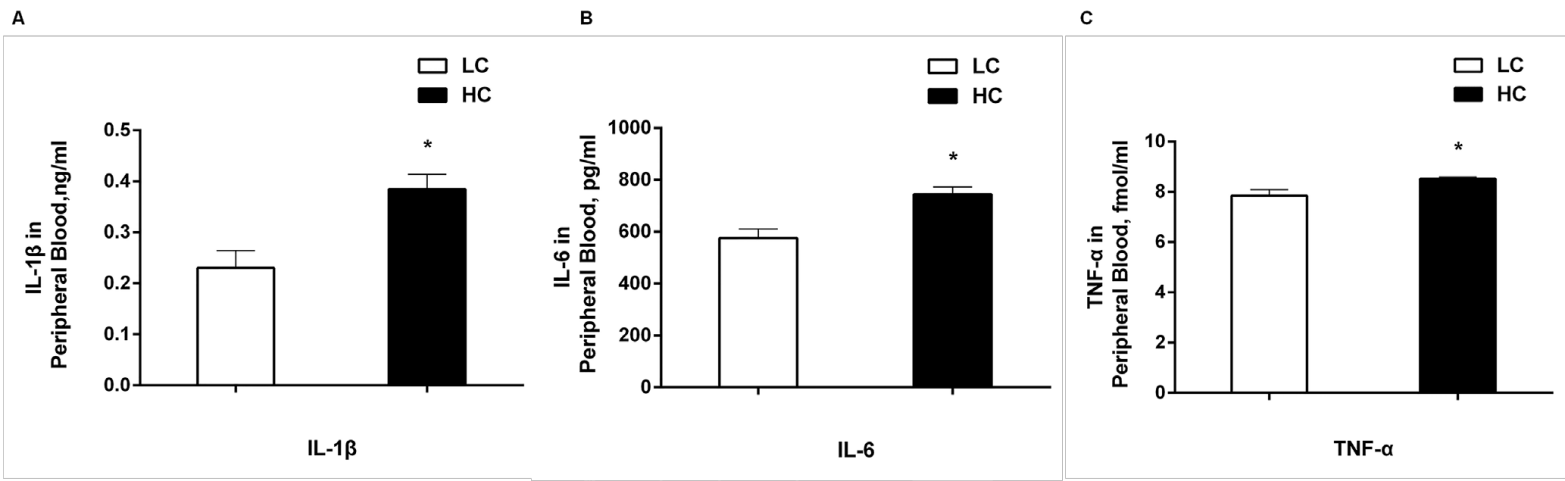
The concentrations of IL-1β **A**., IL-6 **B**., and TNF-α **C**. in peripheral blood of dairy cows from high-concentrate (HC) and low-concentrate (LC) diet. All of three pro-inflammatory cytokines were increased obviously in peripheral blood in HC group compared with LC group. The data are expressed as the mean ± SEM, and asterisks indicate the significant differences between two groups (*P < 0.05, *n* = 6).

The levels of pro-inflammatory cytokines including IL-1β, IL-6 and TNF-α were determined by radioimmunoassay and the results are shown in Figure 6. All of the concentrations of IL-1β (Figure 6A), IL-6 (Figure 6B) and TNF-α (Figure 6C) were significantly higher in the peripheral blood in the HC group compared with the LC group (*P* < 0.01).

## DISCUSSION

In this study, SARA was induced with high-concentrate diet feeding to dairy cows while other researchers used high proportions of grain diet [22–24]. Ruminal pH detection was identified as the only effective method in the current definitions of SARA [5]. There are many different opinions regarding the definitive rumen pH threshold for SARA, such as pH 5.5 [25], 5.2 < pH < 5.6 [2, 26], 5.8 [27] and 6.0 [4] based on the microbial activity in the rumen, the ruminal pH measurement and the integrity of the rumen epithelium. The duration of more than 3 h per day for rumen pH below 5.6 will trigger an inflammatory response during SARA [2]. In our study, ruminal pH below 5.6 that lasted for more than 3 h throughout one day indicated that SARA had been successfully induced.

It has been reported that nearly 60% of ruminal LPS is produced by rapidly growing of gram-negative bacteria [3]. When LPS is released from gram-negative bacteria in the rumen because of the low pH and then translocated into the blood circulation from the gastrointestinal tract, the LPS concentration in the rumen is higher than it would be in healthy cows [8, 11]. Thus, the concentration of free ruminal LPS can also be considered as a determinant of SARA. Andersen et al suggested that free ruminal LPS concentrations increased tenfold during grain diet adaption [11, 12, 22]. However, in recent studies, LPS was not detected in peripheral blood circulation when acute acidosis was induced [22, 28]. It was considered that free ruminal LPS that translocated into the hepatic portal circulation can be detoxified by the liver before reaching the peripheral blood [11, 22]. Meanwhile, large quantities of cytokine receptors appeared in Kupffer cells when foreign pathogens were invading [22]. In our study, the differences of LPS concentration in the portal vein and in the hepatic vein between the two groups, indicated that a mass of free ruminal LPS was translocated into the blood circulation from the digestive tract during SARA, and then detoxified by the liver to prolong the lives of hepatocytes.

Biochemical parameters of TP, ALB, AST, ALT and LDH in plasma are usually measured to evaluate liver function [29–31]. The liver is the primary location for protein synthesized in the body. TP includes ALB and globin with 40-60% being ALB, which synthesis will be affected when 60-80% of liver function is impaired [30, 31]. Therefore, the concentrations of TP and ALB in plasma can be considered as the important indicators of liver healthy. The sensitivity of ALT to hepatocyte damage was much stronger than AST, because, AST existed in all of the organs whereas ALT was confined to hepatocytes [32]. The activity of ALT, AST and LDH in circulation was enhanced in that hepatocyte necrosis occurred or cell membrane permeability increased. The data in Table 3 suggested that hepatocytes were damaged during SARA induced by the HC diet.

Under normal conditions, a small amount of LPS translocated into the liver through the portal vein from the digestive tract, combines with TLR-4 in the Kupffer cells. After that, the combination activates pro-inflammatory cytokines such as IL-1β and TNF-α [33], and causes a systemic inflammatory response. However, histopathologic changes in the liver could be induced through the entrance of superfluous LPS into the liver [34, 35]. It has been reported that Kupffer cells, thrombocytes and leukocytes could be damaged by a high dose of LPS, followed by hepatocyte necrosis [35]. In this study, glycogenated nuclei, inflammatory cells infiltration and hepatocytes swollen happened because of the HC feeding as showed in Figure 3.

**Figure 7:**
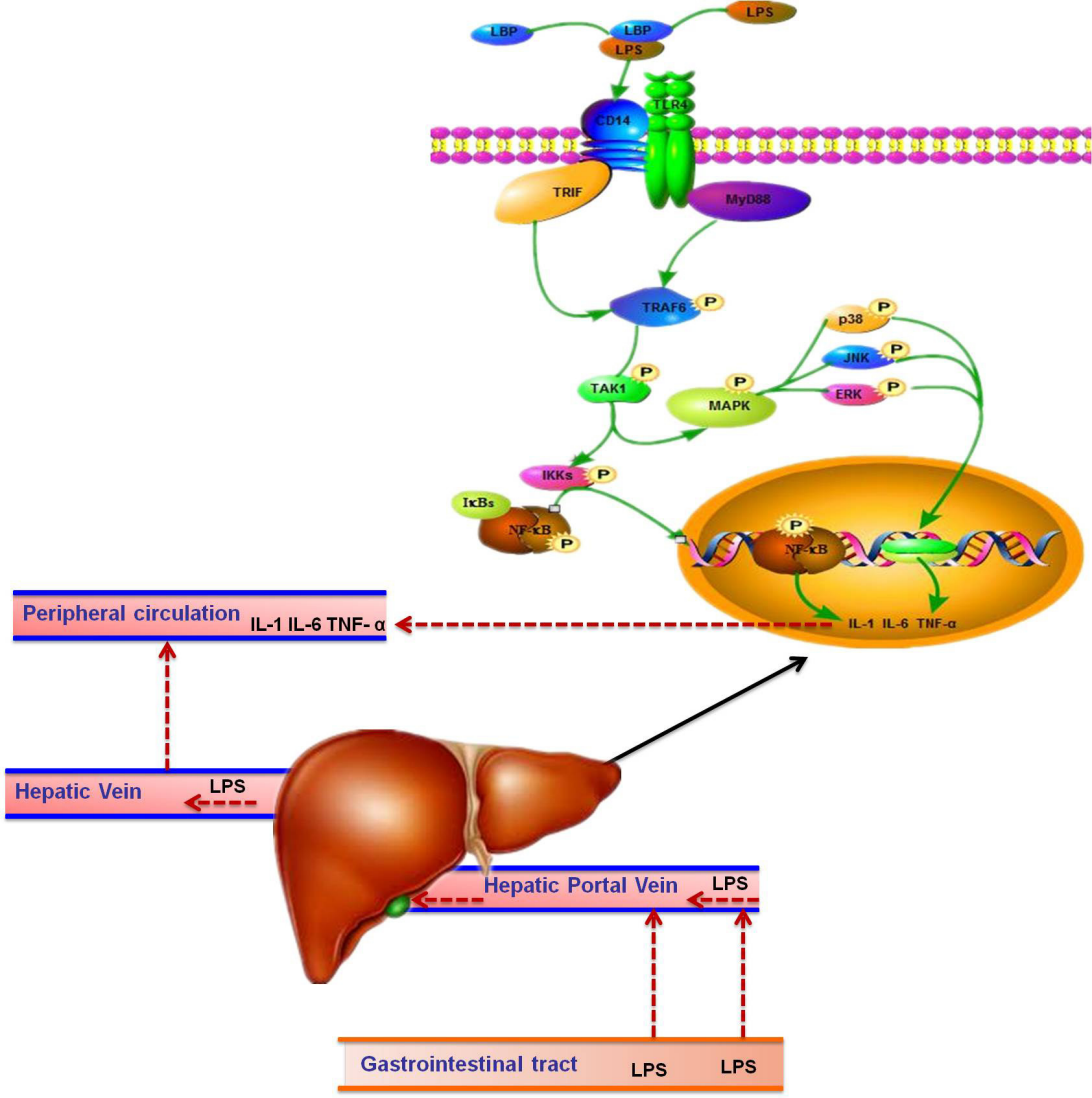
The route of LPS transported to the liver in dairy cows LPS in gastrointestinal tract results from high-concentrate diet was translocated into the liver via portal vein. Then LPS combined with LBP and transported to the hepatocytes surface with the help of CD14, and recognized by TLR4. Subsequently, the complex combined with TRIF or myeloid differentiation factor 88 (MyD88) and activates TRAF6, followed by the activation of Transforming growth factor beta-activated kinase 1 (TAK1). On the one hand, TAK1 activate inhibitor of nuclear factor kappa-B kinases (IKKs) so that NF-κB could be able to escape from IKKs and translocated into the nucleus to stimulate the pro-inflammatory cytokines such as IL-1 and IL-6. On the other hand, TAK1 activate the MAPK to stimulate the pro-inflammatory cytokines production. Those pro-inflammatory cytokines released into the blood circulation and trigger the synthesis of APPs in the liver. As a result, acute phase responses in the body are induced.

SARA can interrupt the rumen epithelium barrier function because of the superfluous volatile fatty acids (VFA) and lactate acid in the rumen [36], which leads to pathogen infiltration and subsequently to liver abscesses and other chronic inflammatory diseases [5, 37, 38]. The presence of pathogens and LPS in blood circulation triggers inflammatory responses. Plasma LPS induces a systemic acute phase response (APR) [18, 22, 36], which is associated with pro-inflammatory cytokines in the liver. It has been established that LBP increases significantly during SARA as a specific marker, facilitates the transfer of LPS to membrane-associated receptors and enhance the immune response [6]. LPS-activated NF-κB in combination with LBP, and triggers the pro-inflammatory mediators of APR, such as IL-1β, IL-6 and TNF-α produced by macrophages or blood monocytes at the site of injury or infection. The cell membrane proteins such as TLR-4 and CD14 combine with LPS [39, 40], and then bind to TIR-domain-containing adapter-inducing interferon-β (TRIF) [41]. Subsequently, macrophages in signaling pathway are activated, which promotes the release of inflammatory cytokines such as IL-1, IL-6 [42] (Figure 7). Those inflammatory cytokines released to blood cirlulation and stimulate liver to synthesize APPs (Figure 7). TLR-4 expression in the liver was inhibited in the HC group in this experiment without significant difference, which contrast to Hirschfeld's study [43]. However, there are also several studies found that the TLR-4 expression in cell membranes decreased during LPS infusion [44, 45]. There is a possibility that hepatocytes excrete endotoxins into the bile from the circulatory system and result in low expression of TLR-4, allowing the hyporesponsiveness of the liver immune system to LPS and remove the endotoxins from the body fluid [46]. Furthermore, the expression of TRAF6, NF-κB, p38 MAPK and ERK MAPK as well as IL-1 increased significantly in the HC group, indicated that the liver contributes to the overall pool of circulating cytokines during inflammation induced by LPS. Moreover, the increase of the mRNA level does not mean expression changes at the protein level. Thus, NF-κB p65 and p38 MAPK, ERK MAPK protein expression, the critical factors that play essential roles in regulating immune responses, were measured. In conventional activation pathway, NF-κB is phosphorylated by IκB kinases-IκKs-which will be subsequently degraded in response to different activators. Then the activated NF-κB complex are liberated and translocated into the nucleus and binds to DNA [44, 47], thereby promoting transcription (Figure 7). The activation of the NF-κB p65 and p38 MAPK were significantly higher in the HC group indicated that the effect on pro-inflammatory responses of LPS was exerted by activating the TLR-4 signaling pathway as showed in Figure 7.

The molecular mechanism of endogenous LPS was also investigated. The levels of IL-1β, IL-6 and TNF-α in peripheral blood increased significantly in the HC group in our experiment. IL-1β involved in host's immune responses against bacteria. IL-6, which production depends on transcription factors such as NF-κB [48] is a multifunction pro-inflammatory cytokine that relate to acute septic shock. As the first of the assayed variables to be affected by LPS, TNF-α plays a critical role in inducing infiltration of neutrophils [9, 49]. The activation of these mediators could be modulated by NF-κB that translocated from the cytoplasm to the nucleus.

In conclusion, LPS derived from the digestive tract to the liver via the portal vein during SARA induced by a high-concentrate diet, which caused hepatocyte impairment, inhibited liver function, enhanced hepatic immune gene expression and activated inflammatory signal pathways.

## MATERIALS AND METHODS

### Ethics statement

The experimental protocols approved by the Animal Care Committee of Nanjing Agricultural University according to the Guidelines for Experimental Animals of the Ministry of Science and Technology (2006, Beijing, China). All surgeries were performed under lidocaine anesthesia, and all efforts were made to minimize the suffering.

### Animals and experimental procedures

Twelve ruminal cannulated lactating Holstein dairy cows (live weight 455 ± 28 kg), installed with jugular, portal and hepatic vein catheters were fed with a LC diet contained 40% concentrate and 60% forages for four weeks. Then these cows were randomly divided into two groups. One was fed with a HC diet contained 60% concentrate and 40% forages as the experimental group. The other one continued with the LC diet as the control group. The ingredient and nutrient composition of the diet for each group are listed in Table 1. Each cow was housed in a bedded pack, fed individually. Both groups were fed with their respective diets for 8 weeks daily at 04:00, 12:00 and 20:00, and had free access to fresh water during the entire experiment.

Sterilized heparin saline (500 IU/mL) was used three times per day to keep the catheters unblocked.

### Sample collection

Rumen fluid samples were taken at 0 h (04:00) post feeding with 1 h interval on sampling day of the eighth week and the pH values were measured immediately.

Blood samples were collected at 0 h (15 min before feeding) with 2 h interval from the jugular, portal and hepatic veins on the sampling day of the eighth week. Plasma was separated by centrifuging the samples in heparinized collection tubes at 1,469 ×g for 15 min and stored at -20°C for further analysis.

Liver tissue samples were collected by biopsy on sampling day of the eighth week, part of them were snap-frozen in liquid nitrogen and stored at -80°C, the others were cut into 1×1cm^2^ pieces and fixed in 4% neutral paraformaldehyde solution for histomorphometric microscopy.

### Measurement of pH and LPS

Rumen pH was measured with a pH-meter. The concentration of LPS in the plasma of portal and hepatic veins was determined by Limulus Amebocyte Lysate (LAL) assay with a diazo coupling reagent (Chinese Horseshoe Crab Reagent Manufactory Co., Ltd., Xiamen, China), which has a minimum detectable concentration of 0.01 EU/mL. The procedures followed the manufacturer's instructions.

### Histopathologic and biochemical parameters analysis

Liver tissue sections were made from paraffin-embedded tissues. The sections were stained with hematoxylin and eosin, and the histopathologic changes were observed under a light microscopy (Nikon ECLIPSE 50i) and the micrographs were taken with a digital camera (Nikon Digital Sight DS-Fi1, Nikon Corporation, Minato-ku, Tokyo, Japan) and NIS Elements F 3.0 (Nikon Corporation, Minato-ku, Tokyo, Japan) image acquisition software. The hepatocytes damages were conducted using a score system described by Kleiner et al [50]. Briefly, the damage score consisted of scores for glycogenated nuclei (graded 0-1, from none or rare to many continuous patches), liver cells ballooning injury (graded 0-2, from absent to severe ballooning injury) and inflammatory cells filtrate (graded 0-3, from absent to transmural). Three tissue sections from each animal were coded and examined by two blinded observers to prevent observer bias.

Biochemical parameters such as Total protein, Albumin, Aspartate aminotransferase, Alanine aminotransferase and Lactate dehydrogenase were determined using a biochemical analyzer (SPOTCHEM™EZ SP-4430, Arkray, Kyoto, Japan).

### Real-time quantitative PCR

Total RNA was extracted from liver tissues (*n* = 6) with Trizol (Takara, Dalian, China) according to the manufacturer's protocols, then the concentration was quantified by measuring the absorbance using spectrophotometer (Nannodrop ND-2000, (Thermo Fisher Scientific Inc., Waltham, United States). The cDNA was synthesized by reverse transcription (Cat. RR036A, Takara) with specific oligo (dT) primers for mRNAs (Table 2). Primers for target genes were designed with Primer Premier Software 5.0 (Premier Biosoft International, USA). Then, RT-qPCR was performed with the ABI 7300 instrument (Applied Biosystems, Foster City, CA, USA) to determine the relative copy numbers of different mRNAs. The amplifications were performed with the following protocols: 95°C for 30 s, followed by 40 cycles composed of 5 s at 95°C and 31 s at 60°C, 95°C for 15 s, 60°C for 1min and 95°C for 15 s. The SYBR Premix EX Taq™ 144 kit (Cat. DRR420A, Takara) was used to amplify the target segment of cDNA, and melting curves were performed to ensure a single specific PCR product for each gene. Gene expression was normalized to GAPDH mRNA levels as a housekeeping gene and the data were analyzed according to the 2^-ΔΔCT^ method.

### Western blot analysis

Total protein was extracted using RIPA Lysis Buffer (Cat. SN338, Sunshine Biotechnology Co., Ltd, Nanjing, China) from liver tissue took from −80°C. The protein concentration was determined by BCA assay (Pierce, Rockford, USA). Each protein sample (100 mg) was added to loading buffer and denatured at 100°C for 5 min before being subjected to 10% sodium dodecyl sulfate-polyacrylamide gel electrophoresis (SDS-PAGE), the separated proteins were then transferred to nitrocellulose membranes (Bio Trace, Pall co., USA). Western blot analysis for NF-κB (p65, sc-21014, SAB, USA, 1:500 dilution; Phospho-p65, sc-11014, SAB, USA, 1:500 dilution), p38 (Phospho-p38 MAPK, 4511s, CST, Shanghai, China, 1:1000 dilution; p38 MAPK, 8690s, CST, Shanghai, China, 1:1000 dilution) and ERK 1/2 (Phospho-p44/42 MAPK, 4370s, CST, Shanghai, China, 1:1000 dilution; p44/42 MAPK, 4695s, CST, Shanghai, China, 1:1000 dilution) were implemented with the primary antibody and corresponding HRP-conjugated secondary antibody, β-tubulin (Cat. SAM1002, Sunshine Biotechnology Co., Ltd, Nanjing, China, 1:3,000 dilution) and GAPDH (Cat. sc-32233, Santa Cruz Biotechnology Co., Ltd, Shanghai, china, 1:200 dilution) were used as reference proteins for normalization, respectively. The antibody conjugated HRP was detected by enhanced chemiluminescence detection (Super Signal West Pico Trial Kit, Pierce, USA) and quantified using a VersaDoc 4000 MP system (Bio-Rad). The levels of NF-κB were expressed as fold changes relative to the average value in the LC group.

### Radioimmunoassay

A gamma Radioimmunoassay Counter (Shanghai Hesuo Rihuan Photoelectric Instrument Co., Ltd, China) was used to determine the concentrations of IL-1β, IL-6 and TNF-α in peripheral blood with radioimmunoassay kits ( IL-1β, C09DJB; IL-6, C12DJB; TNF-α, C06PJB; Beijing North Institute of Biological Technology, Beijing, China).

### Statistical analysis

The data are expressed as the mean ± SEM. The concentration of LPS and rumen pH were analyzed with SAS mixed model using sampling time as repeated measurements. The expression of inflammatory cytokines and proteins was determined with the paired samples t-test in SPSS 20.0. Differences were considered significant when *P* < 0.05.
